# Influence of root width and dentin wall thickness evaluated by endoscopy upon the outcome of periapical surgery. A cohort study

**DOI:** 10.4317/medoral.25314

**Published:** 2022-06-05

**Authors:** Pablo Glera-Suárez, Antonio Pallarés-Serrano, David Soto-Peñaloza, Beatriz Tarazona-Alvarez, Miguel Penarrocha-Diago, David Penarrocha-Oltra

**Affiliations:** 1 DDS, Master in Oral Surgery and Implant Dentistry, Department of Stomatology, University of Valencia Medical and Dental School, Valencia, Spain; 2 DDS, PhD, Master in Oral Surgery and Implant Dentistry, Department of Stomatology, University of Valencia Medical and Dental School, Valencia, Spain; 3 DDS, PhD, Assistant Professor, Orthodontic Unit, Department of Stomatology, University of Valencia Medical and Dental School, Valencia, Spain; 4 MD, PhD, DDS, Professor and Chairman, Oral Surgery Unit, Department of Stomatology, University of Valencia Medical and Dental School, Researcher at the IDIBELL Institute, Barcelona, Spain; 5 DDS, PhD, Professor, Oral Surgery Unit, Department of Stomatology, University of Valencia Medical and Dental School, Valencia, Researcher at the IDIBELL Institute, Barcelona, Spain

## Abstract

**Background:**

An analysis was made of the correlation between root width, the thickness of the remaining dentinal wall as determined by endoscopy, and the outcome of periapical surgery.

**Material and Methods:**

A retrospective cohort study was carried out involving patients subjected to periapical surgery between 2017 and 2019 at the University of Valencia (Valencia, Spain).

One year after surgery, cone-beam computed tomography (CBCT) was used to evaluate healing against the preoperative volumes. The maximum root width was measured on the postoperative CBCT scan at the apical section of the treated root. This measurement was transferred to the intraoperative endoscopic image, where the minimum root width, peripheral dentin thickness, and minimum dentin thickness were recorded. Root measurements, and the position (maxillary or mandibular) and type of tooth (roots of incisors, canines, premolars or molars) were further correlated to periapical surgery outcome.

**Results:**

A total of 51 patients, comprising 52 teeth and 62 roots, were included in the study. The mean measurements were: maximum root width (4.13±0.84 mm), minimum root width (2.46±0.72 mm), peripheral dentin thickness (0.77±0.2 mm) and minimum dentin thickness (0.4±0.2 mm). The success rate was 82.2%. Premolar roots showed a greater minimum dentin thickness (0.58±0.25 mm) (*p*<0.003) than incisor roots. No significant association was found between the different measurements and the healing rate at one year, though the roots that failed to heal showed smaller minimum dentin thickness values than the roots that healed correctly. The position and type of tooth did not influence healing outcome.

**Conclusions:**

The root width and thickness of the remaining dentin wall did not significantly influence healing. However, the roots that failed to heal showed smaller minimum dentin thickness values than the roots that healed correctly.

** Key words:**Endodontic surgery, endoscope, dentin walls.

## Introduction

The aim of periapical surgery is to treat persistent chronic periapical periodontitis in those cases where orthograde root canal retreatment is not possible (1). The technique has evolved over the years, and surgical field illumination and magnification systems have been introduced, such as the microscope and endoscope, resulting in periapical surgery success rates of over 90% (2). The endoscope allows accurate inspection at root resection level (2,3). It offers advantages such as the identification of root tips, possible root fractures, canals not sealed through orthograde filling, and the joining of canals (isthmuses) (4). Likewise, the instrument allows confirmation of proper sealing of the retrograde apical cavity (5,6). Compared to the microscope, the rigid endoscope is associated to lower cost, and the learning curve is easier (7).

During periapical surgery, it has been described that a 3-mm apicoectomy should be performed to eliminate all apical ramifications and lateral canals, and to avoid reinfection of the periapical area and therefore recurrence of the lesion (8-10). The root section must be perpendicular to the longitudinal axis of the tooth (11), and it has been suggested that the retrograde cavity should have a depth of 3 mm and follow the original path of the root canal (12). In vitro studies have demonstrated that if the retrograde cavity is performed with beveling, microleakage occurs through the area where the remaining dentin wall is thinner (13,14).

To date, only one clinical study, published by von Arx *et al*. (15), has analyzed the *in vivo* mean thickness of the dentin wall remaining around the retrograde filling. These authors performed four measurements in the buccolingual and mesiodistal planes of the roots using cone-beam computed tomography (CBCT) at 12 months after surgery. However, the diameter of the remaining root surface after apical resection, and the zone of the dentin wall with the smallest dentin thickness, are possible periapical surgery prognostic factors that have not yet been clinically evaluated.

The present study was carried out to analyze the relationship between the root diameter and thickness of the remaining dentin wall at root surface level following apicoectomy and the periapical surgery healing rate at one year, using the control CBCT scan made one year after the operation and the intraoperative endoscopic images obtained after retrograde filling.

## Material and Methods

- Study design

A retrospective cohort study was conducted from September 2017 to December 2019 at the Oral Surgery and Implantology Unit (Department of Stomatology, University of Valencia Medical and Dental School, Valencia, Spain). The study was conducted in abidance with the Declaration of Helsinki (1975 as revised in 2013) regarding biomedical research in human subjects, and was approved by the Ethics Committee of the University of Valencia (Protocol ref.: 1126870). The present manuscript is reported according to the Strengthening the Reporting of Observational Studies in Epidemiology (STROBE) statement for cohort studies (www.strobe-statement.org).

- Sample selection

The inclusion criteria were healthy patients without serious systemic diseases or functional limitations and with sTable periodontal conditions subjected to endodontic microsurgery using ultrasonic tips, a rigid endoscope to obtain high-magnification intraoperative photographs, mineral trioxide aggregate (MTA) (Dentsply®, Tulsa Dental Specialties, Tulsa, OK, USA) for retrograde filling, and CBCT before surgery and after one year of follow-up. The exclusion criteria were patients failing to come to the control visits, through-and-through lesions, apicomarginal defects, cases in which bone regeneration of the defect was performed, and poor quality endoscopic images or images with artifacts precluding evaluation of the study variables.

- Surgical technique

In all cases, local infiltration anesthesia was provided with 4% articaine and epinephrine (1:100,000) (Inibsa®; Llica of Vall, Barcelona, Spain), and all surgeries were performed using a dental operating microscope (Möller® Dental 300, Wedel, Germany) and a rigid endoscope with 30° forward view and 2.7 mm in diameter (HOPKINS® optics model 7207 BA, Karl Storz-Endoskope®, Tuttlingen, Germany) as magnification and illumination devices. Paramarginal or submarginal incisions were performed. After mucoperiosteal flap release, an ostectomy was carried out using round 0.27 mm tungsten carbide drills (Jota, Switzerland) mounted in a 1:1 handpiece (W&H®, Bürmoos, Austria) under irrigation with sterile saline solution. Hemostasis was secured with Expasyl™ (Pierre Rolland, Merignac, France) or sterile polytetrafluoroethylene (PTFE) strips (16).

**Figure 1:**
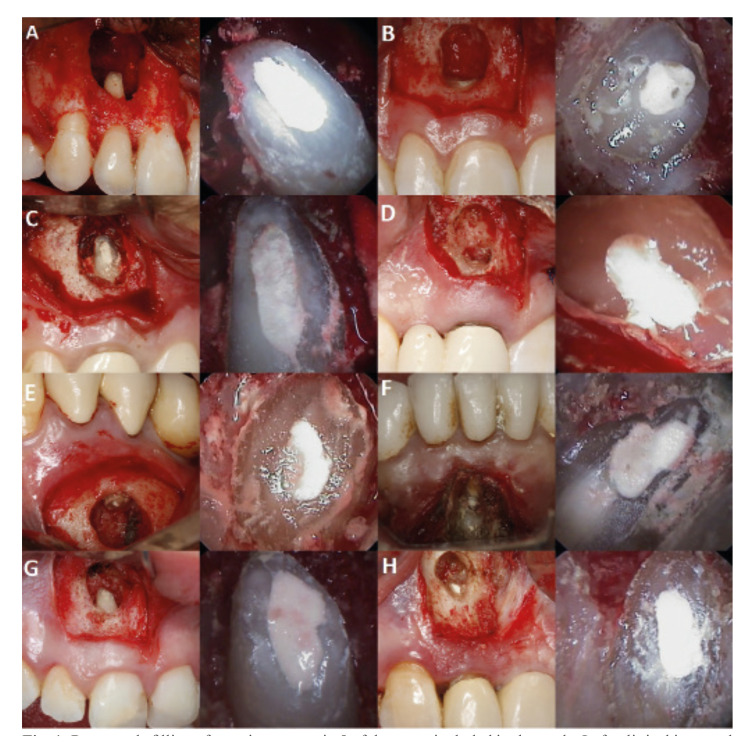
Retrograde filling after apicoectomy in 8 of the cases included in the study. Left: clinical intraoral view. Right: endoscopic view.

The apical portion was resected 3 mm, as perpendicular as possible to the longitudinal axis of the tooth, and the root end surface was inspected with the endoscope. The retrograde cavities were then prepared 3 mm in depth with ultrasonic retrotips (Piezomed®, W&H, Bürmoos, Austria), followed by retrofilling with MTA (Dentsply®, Tulsa Dental Specialties, Tulsa, OK, USA). Intraoperative photographs were obtained using the rigid endoscope with the highest possible magnification. Tension-free flap closure was performed using 6/0 suture material (Polinyl®, Sweden & Martina, Carrare, Italy). Fig. 1 shows the clinical and endoscopic views of 8 of the 62 roots included in the study sample.

- Radiographic assessment

The preoperative and follow-up CBCT volumes were taken using a Planmeca® ProMax 3D Classic device (Planmeca, Helsinki, Finland). The field of view (FOV) was either 5x5 cm (voxel size 0.010 mm) or 5x8 cm (voxel size 0.150 mm). The exposure parameters were 6.3 or 8.0 mA for medium and large size skulls at 90kV, with an exposure time of 12 and 15 seconds for 180° rotation, respectively.

**Figure 2:**
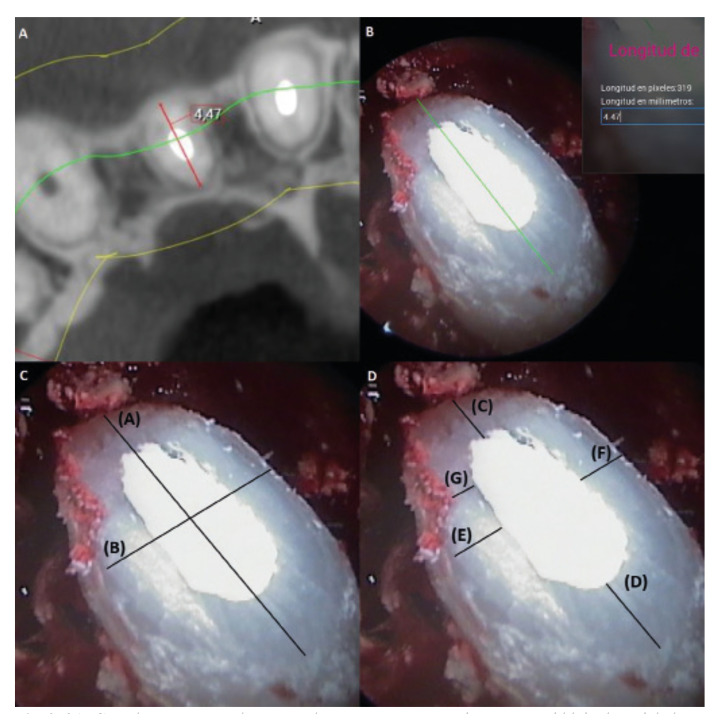
2A: Cone-beam computed tomography measurement: maximum root width in the axial plane. The CBCT image corresponds to Figure 1A; 2B: Calibration of the endoscopic image from the measurement of the maximum root width obtained in the CBCT; 2C: Endoscopic measurements. (A): maximum root width; (B): minimum root width; 2D: Endoscopic measurements. (C, D, E, F): measurements of peripheral dentin thickness; (G): minimum dentin thickness.

The 12-month follow-up CBCT volumes were assessed twice by two authors (P.G.S. and D.S.P.) not involved in the surgery procedures, at an interval of four weeks. Maximum root width (buccal-lingual/palatal) in the axial plane was recorded using the Romexis 5.2.1.R application (Planmeca, Helsinki, Finland) (Fig. 2). The means of the two measurements of the two observers were pooled for the final analysis and further calibration of the endoscopic images in duplicate (P.G.S. and A.P.S.).

- Collection of endoscopic images

The surface of the resected root apex was inspected intraoperatively with a rigid endoscope with 30° forward view and 2.7 mm in diameter (HOPKINS® optics model 7207 BA, Karl Storz-Endoskope®, Tuttlingen, Germany). Images were captured and processed using a documentation device providing 5600 K daylight coloration, with a 50 W (1000 lumens) halogen lamp illumination source (TELE PACK™ PAL Control Unit 200430-20, Karl Storz-Endoskope®) and using a digital camera with Parfocal Zoom Lens, f=25-50 mm (2x) (TELECAM® PAL color system, Karl Storz-Endoskope®).

Images were coded and exported to a PDF file by a third author not involved in appraisal of the images (B.T.A.), to implement blinding of the evaluators. Then, two authors (P.G.S. and A.P.S.) assessed the endoscopic images for validity in terms of quality (sharpness) and to exclude those with the presence of artifacts (e.g., incomplete root apex visibility, blurred or foggy images) capable of impeding adequate evaluation. Disagreements were resolved by consensus with a third author (D.S.-P.). For this procedure, the images were visualized on a Full HD monitor (1920 x 1080 pixels) under subdued lighting (iMac Pro, Apple, Cupertino, CA, USA). The appraised images were categorized as adequate or inadequate according to the abovementioned criteria. The level of agreement at this stage was determined based on the intraclass correlation coefficient (ICC), as previously described (16).

- Calibration of endoscopic images and examiners

Calibration of the rigid endoscope images was performed in duplicate (P.G.S. and A.P.S.), and involved transfer of the maximum root width measurement obtained from the CBCT slices to the intraoperative endoscopic capture, with use of the Romexis 5.2.1.R application (Fig. 2). From this point, the following study parameters were defined (Fig. 2):

1) Maximum root width: buccolingual/palatal root width (A).

2) Minimum root width: mesiodistal root width (B).

3) Peripheral dentin thickness: mean dentin thickness peripheral to the retrograde filling. For this purpose, four measurements were made on the dentin wall – two of them (C) and (D) in the buccolingual/palatine plane, and the other two (E) and (F) in the mesiodistal plane. The mean dentin thickness (peripheral dentin thickness) was calculated from the mean of these four measurements.

4) Minimum dentin thickness (G): measurement made on the narrowest zone of the dentin wall.

The repeatability of the measurements taken from the endoscopic images was calibrated based on two series of measurements at an interval of one week, using 5 cases with apicomarginal defects that were deemed to be excluded from the study. The measurements were pooled, and a mean was calculated for analysis.

The level of agreement of the linear measurements between reviewers for calibration of both the CBCT and endoscopic images was obtained by calculating the ICC, with interpretation according to the Landis and Koch scale (17).

- Study outcomes

The main study variables were the maximum (buccolingual/palatine) and minimum (mesiodistal) root width, peripheral dentin thickness and minimum dentin thickness around the retrograde filling cavity, and healing outcome one year after surgery. As secondary variables, we evaluated the type of tooth (roots of incisors, canines, premolars and molars), and tooth position (maxillary or mandibular).

- Healing assessment

Healing was independently evaluated for each root, considering the clinical and radiographic parameters of the CBCT volumes at the 12-month postoperative recall. Pain, sensitivity in response to palpation or percussion, inflammation and the presence of a fistula were categorized as unsatisfactory healing. The CBCT sagittal plane was parallel to the mesiodistal long axis of the tooth; the coronal plane was aligned with the root canal; and both planes passed through the middle of the resected root end.

**Figure 3:**
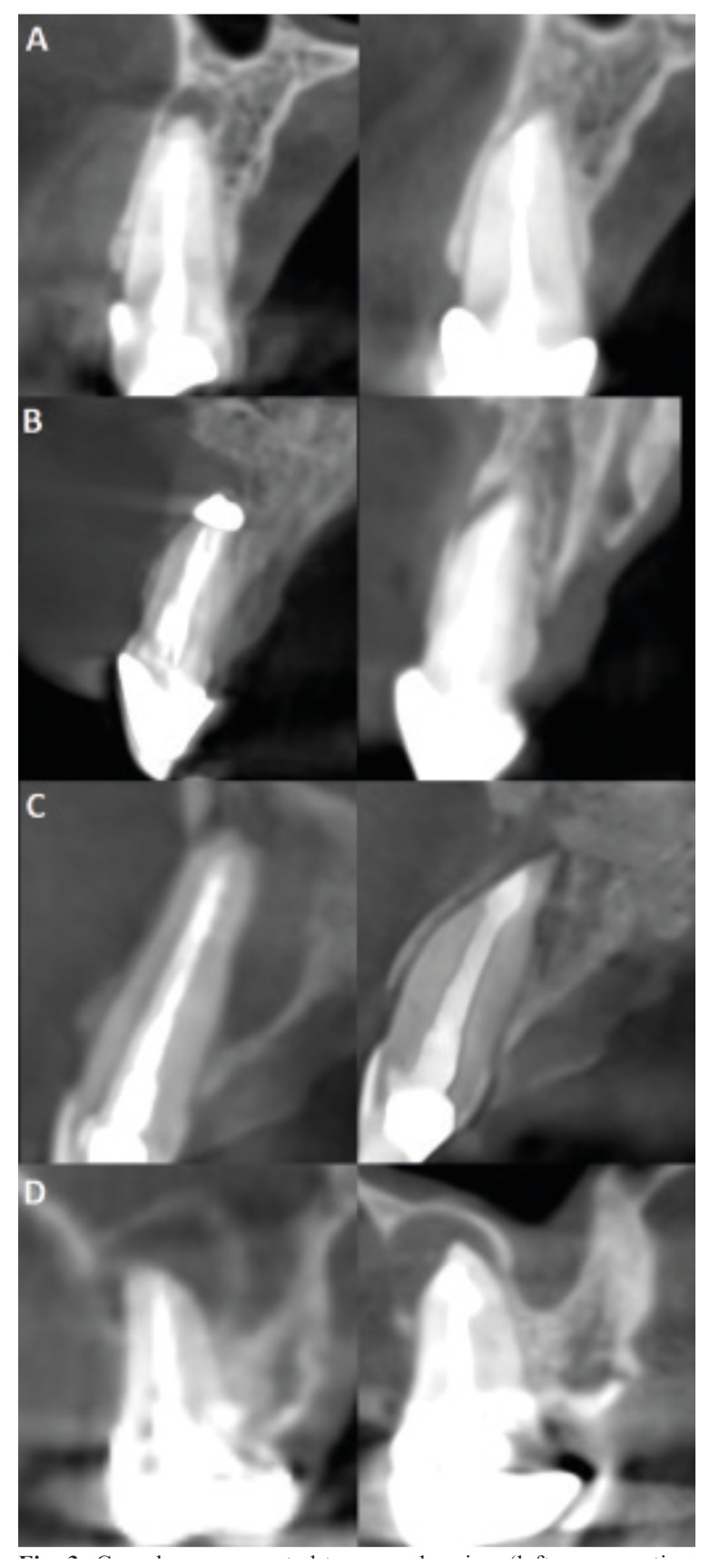
Cone-beam computed tomography view (left: preoperative; right: postoperative) of four of the cases included in the study, showing the four healing subgroups according to the modified PENN 3D criteria: A) complete healing, B) limited healing, C) uncertain healing, D) unsatisfactory healing.

Radiographic healing status was independently evaluated by two calibrated observers (P.G.-S., A.P.-S.) and categorized into four subgroups, based on the modified PENN 3D criteria (18), as follows: complete, limited, uncertain, unsatisfactory. The results obtained were dichotomized into healed and non-healed categories. Cases classified as complete or limited healing were regarded as healed, whereas those classified as uncertain or unsatisfactory healing were grouped as non-healed. Images were inspected under standardized conditions. Magnification tools were used when deemed necessary. A kappa (k) value for agreement was calculated, and discrepancies were resolved by discussion with a third advisor (D.S.-P.). The characteristics of the healing criteria are depicted in Fig. 3.

- Statistical analysis

A descriptive analysis was made of the study variables, with determination of the corresponding 95% confidence interval (95%CI) for linear parameters. An inferential analysis was performed of the linear parameters, with exploration of the association between treatment outcome and the independent variables. Generalized Estimating Equation (GEE) models were used to analyze the probability of treatment failure according to each of the independent variables. Unadjusted odds ratios (ORs) were estimated, and the effect was measured using the Wald Chi-squared statistic.

The level of statistical significance was established as 5% (α=0.05). Absolute inter-rater agreement was evaluated using a two-way mixed-effects model for the means of (k=2) evaluators, and interpreted as proposed by Shrout and Fleiss (19). Mean estimations along with 95% confidence intervals (95%CI) were reported for each ICC, with interpretation as follows: poor < 0.50; fair 0.50-0.75; good 0.75-0.90; and excellent > 0.90.

## Results

- Sample selection and sample features

Of the initial sample of 70 patients, 8 who failed to come to the one-year follow-up visit were excluded, in the same way as four with apicomarginal defects, two with through-and-through lesions, and two in which bone regeneration of the defect was performed due to the large size of the lesion.

Furthermore, after analyzing the endoscopic images of the 54 potential eligible patients, three were excluded due to poor quality endoscopic images or images with artifacts precluding evaluation of the study variables. Inter-rater consistency was almost perfect (ICC = 0.98, *p*=0.001).

Fifty-one patients were thus finally included in the study, comprising 52 teeth and 62 roots. Of these subjects, 31 were women (60.7%) and 20 men (39,3%), with a mean age of 47.0 ± 14.9 years (range 18-76). Regarding the 62 included roots, 41 were maxillary (66.1%) and 21 mandibular (33.9%); 21 belonged to incisors (33.9%), 6 to canines (9.7%), 13 to premolars (20.9%) and 22 to molars (35.5%) (Table 1).

- Root width and dentin thickness

The mean maximum and minimum root widths were 4.1±0.8 mm and 2.5±0.7 mm respectively. With regard to the measurements made, inter-rater agreement proved to be almost perfect for both parameters (ICC = 0.92, 95%CI [0.88; 0.94]) and (ICC = 0.96, 95%CI [0.93; 0.99]). With regard to the position and type of tooth, no significant differences in root dimensions were observed.

Peripheral dentin thickness and minimum dentin thickness were 0.77±0.2 mm and 0.4±0.2 mm respectively. The ICC for inter-rater agreement was almost perfect for both measurements (ICC = 0.98, 95%CI [0.96; 1.00]) and (ICC=0.90, 95%CI [0.87; 0.93]). No minimum dentin thickness values of ≥ 1 mm were obtained in any of the cases included in the study. Premolar roots showed a greater minimum dentin wall thickness (0.58±0.25) than incisor roots (*p*<0.003). With regard to tooth position, no significant differences in root dimensions were observed. The position and type of teeth are described in Table 1.

- Healing outcomes and inferential analysis

Complete healing was observed in 54.8% of the roots, limited healing in 27.4%, uncertain healing in 11.3%, and unsatisfactory healing in 6.5%. One of the cases presented an active fistula with inflammation of the zone, and was thus classified as unsatisfactory healing.

Based on dichotomization into healed and non-healed categories, the final success rate at one year of follow-up was 82.2%. The different measurements made and their distribution among healing categories are reported in Table 2.

**Figure 4:**
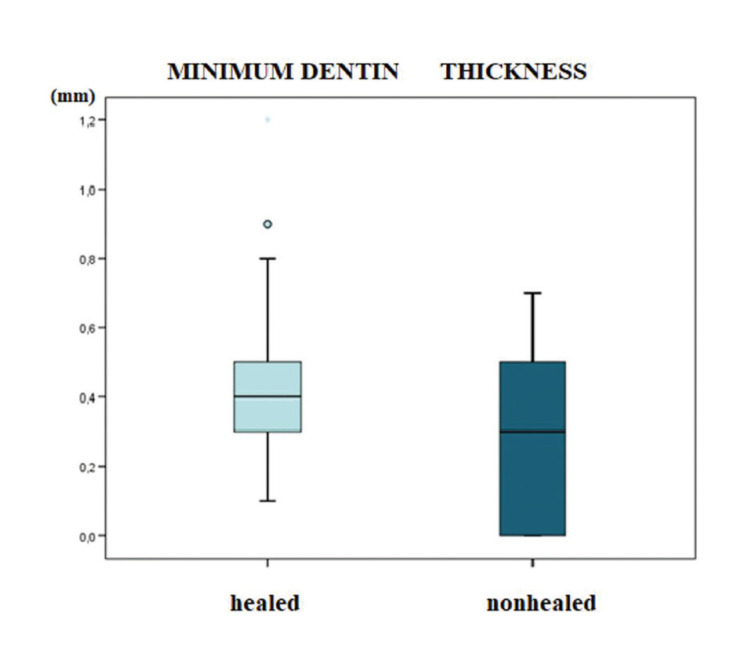
Box plot. Relationship between minimum dentin wall thickness and healing.

No statistically significant association was observed between the study variables (root and dentinal wall measurements, position and type of tooth) and the periapical surgery healing rate at one year (Table 3). Although no significant correlation was found between minimum dentin thickness and healing of the lesion (*p*>0.05), those roots that failed to heal were seen to have smaller minimum dentinal thickness values than the roots that healed correctly (Fig. 4).

**Table 1:**
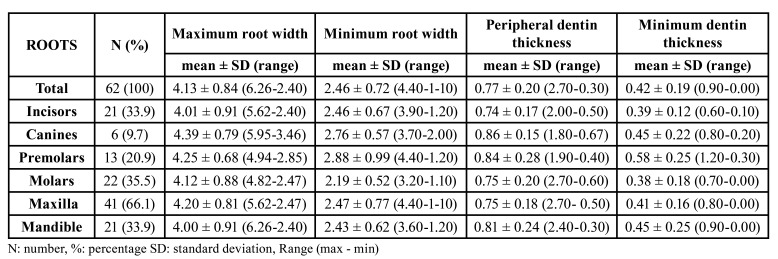
Root dimensions (mm) according to the type and position of the root subjected to periapical surgery.

**Table 2:**
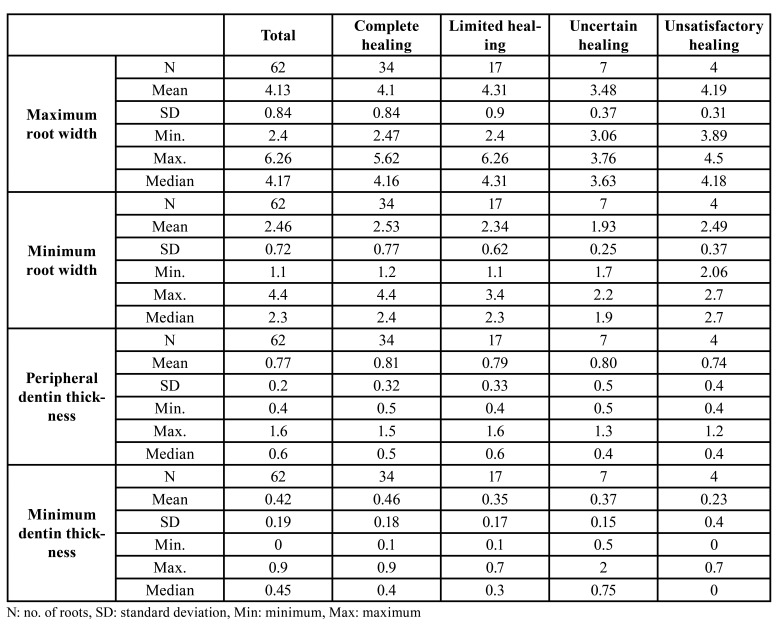
Association between root dimensions (mm) and healing after periapical surgery.

**Table 3:**
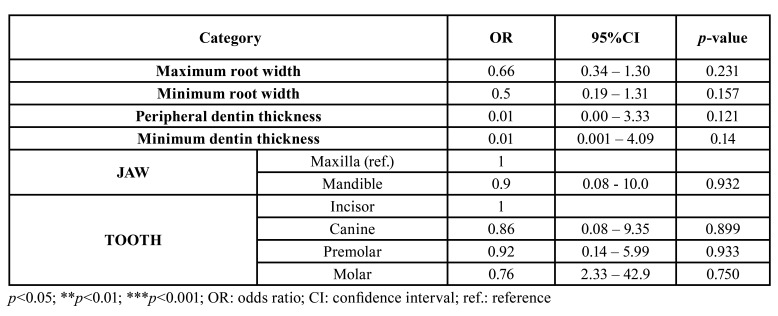
Association between healing and independent covariables. Linear Generalized Estimating Equation (GEE) models of simple binary logistic regression analysis.

## References

[1] Kim S, Kratchman S (2006). Modern endodontic surgery concepts and practice: a review. J Endod.

[2] Pallarés-Serrano A, Glera-Suarez P, Soto-Peñaloza D, Peñarrocha-Oltra D, von Arx T, Peñarrocha-Diago M (2020). The use of the endoscope in endodontic surgery: A systematic review. J Clin Exp Dent.

[3] von Arx T, Hunenbart S, Buser D (2002). Endoscope and videoassisted endodontic surgery. Quintessence Int.

[4] Bahcall JK, Barss J (2003). Orascopic visualization technique for conventional and surgical endodontics. Int Endod J.

[5] Taschieri S, Del Fabbro M, Testori T, Francetti L, Weinstein R (2006). Use of a surgical microscope and endoscope to maximize the success of periradicular surgery. Pract Proced Aesthet Dent.

[6] Del Fabbro M, Taschieri S (2010). Endodontic therapy using magnification devices: A systematic review. J Dent.

[7] von Arx T, Montagne D, Zwinggi C, Lussi A (2003). Diagnostic accuracy of endoscopy in periradicular surgery - a comparison with scanning electron microscopy. Int Endod J.

[8] Pop I (2003). Oral surgery: part 2. Endodontic surgery. Br Dent J.

[9] Kim S, Kratchman S (2006). Modern endodontic surgery concepts and practice: A review. J Endod.

[10] Chong BS, Rhodes JS (2014). Endodontic surgery. Br Dent J.

[11] Serrano-Giménez M, Sánchez-Torres A, Gay-Escoda C (2015). Prognostic factors on periapical surgery: A systematic review. Med Oral Patol Oral Cir Bucal.

[12] Martí Bowen E, Peñarrocha M (2006). An update in periapical surgery. Med Oral Patol Oral Cir Bucal.

[13] Gutmann JL, Pitt Ford TR (1993). Management of the resected root end: a clinical review. Int Endod J.

[14] Post LK, Lima FG, Xavier CB, Demarco FF, Gerhardt-Oliveira M (2010). Sealing ability of MTA and amalgam in different root-end preparations and resection bevel angles: an in vitro evaluation using marginal dye leakage. Braz Dent J.

[15] von Arx T, Marwik E, Bornstein MM (2019). Apical Surgery: Effects of Dimensions of Root-End Fillings and Peripheral Root Dentine on the Healing Outcome. Eur Endod J.

[16] Peñarrocha-Oltra D, Soto-Peñaloza D, Peñarrocha-Diago M, Cervera-Ballester J, von Arx T, Peñarrocha-Diago M (2019). Hemostatic Agents in Endodontic Surgery: A Randomized Controlled Pilot Study of Polytetrafluoroethylene Strips as an Adjunct to Epinephrine Impregnated Gauze Versus Aluminum Chloride. J Endod.

[17] Landis JR, Koch GG (1977). An application of hierarchical kappa-type statistics in the assessment of majority agreement among multiple observers. Biometrics.

[18] Schloss T, Sonntag D, Kohli MR, Setzer FC (2017). A comparison of 2- and 3-dimensional healing assessment after endodontic surgery using cone-beam computed tomographic volumes or periapical radiographs. J Endod.

[19] Shrout PE, Fleiss JL (1979). Intraclass correlations: uses in assessing rater reliability. Psychol Bull.

[20] Cotti E, Vargiu P, Dettori C, Mallarini G (1999). Computerized tomography in the management and follow-up of extensive periapical lesion. Endod Dent Traumatol.

[21] Lin CP, Chou HG, Kuo JC, Lan WH (1998). The quality of ultrasonic root-end preparation: A quantitative study. J Endod.

[22] Roy R, Chandler NP, Lin J (2008). Peripheral dentin thickness after root-end cavity preparation. Oral Surg Oral Med Oral Pathol Oral Radiol Endod.

[23] Tawil PZ, Saraiya VM, Galicia JC, Duggan DJ (2015). Periapical microsurgery: the effect of root dentinal defects on short- and long-term outcome. J Endod.

[24] Abedi HR, Van Mierlo BL, Wilder-Smith P, Torabinejad M (1995). Effects of ultrasonic root-end cavity preparation on the root apex. Oral Surg Oral Med Oral Pathol Oral Radiol Endod.

[25] Frank RJ, Antrim DD, Bakland LK (1996). Effect of retrograde cavity preparations on root apexes. Endod Dent Traumatol.

[26] Gondim E Jr, Gomes BP, Ferraz CC, Teixeira FB, Souza-Filho FJ (2002). Effect of sonic and ultrasonic retrograde cavity preparation on the integrity of root apices of freshly extracted human teeth: scanning electron microscopy analysis. J Endod.

[27] Khabbaz MG, Kerezoudis NP, Aroni E, Tsatsas V (2004). Evaluation of different methods for the root-end cavity preparation. Oral Surg Oral Med Oral Pathol Oral Radiol Endod.

[28] De Bruyne MA, De Moor RJ (2005). SEM analysis of the integrity of resected root apices of cadaver and extracted teeth after ultrasonic root-end preparation at different intensities. Int Endod J.

[29] Tobon-Arroyave SI, Restrepo-Perez MM, Arismendi-Echavarria JA, Velasquez-Restrepo Z, Marin-Botero ML, Garcia-Dorado EC (2007). Ex vivo microscopic assessment of factors affecting the quality of apical seal created by root-end fillings. Int Endod J.

